# Quality improvement in small office settings: an examination of successful practices

**DOI:** 10.1186/1471-2296-10-14

**Published:** 2009-02-09

**Authors:** Daniel Wolfson, Elizabeth Bernabeo, Brian Leas, Shoshanna Sofaer, Gregory Pawlson, Donna Pillittere

**Affiliations:** 1 American Board of Internal Medicine Foundation, Philadelphia, PA, USA; 2 Quality Research, American Board of Internal Medicine, Philadelphia, PA, USA; 3 Department of Health Policy, Thomas Jefferson University, Philadelphia, PA, USA; 4 School of Public Affairs, Baruch College, New York, NY, USA; 5 National Committee for Quality Assurance, Washington, DC, USA

## Abstract

**Background:**

Physicians in small to moderate primary care practices in the United States (U.S.) (<25 physicians) face unique challenges in implementing quality improvement (QI) initiatives, including limited resources, small staffs, and inadequate information technology systems 23,36. This qualitative study sought to identify and understand the characteristics and organizational cultures of physicians working in smaller practices who are actively engaged in measurement and quality improvement initiatives.

**Methods:**

We undertook a qualitative study, based on semi-structured, open-ended interviews conducted with practices (N = 39) that used performance data to drive quality improvement activities.

**Results:**

Physicians indicated that benefits to performing measurement and QI included greater practice efficiency, patient and staff retention, and higher staff and clinician satisfaction with practice. Internal facilitators included the designation of a practice champion, cooperation of other physicians and staff, and the involvement of practice leaders. Time constraints, cost of activities, problems with information management and or technology, lack of motivated staff, and a lack of financial incentives were commonly reported as barriers.

**Conclusion:**

These findings shed light on how physicians engage in quality improvement activities, and may help raise awareness of and aid in the implementation of future initiatives in small practices more generally.

## Background

After the Institute of Medicine (IOM) published To Err is Human,[1] numerous medical groups, health plans, and clinical organizations engaged in strategies to facilitate quality improvement (QI) in healthcare in the U.S. Central to these efforts is the recognition that improvement requires performance measurement, yet measurement alone is not sufficient. This linkage of performance measurement to improvement is well noted in both the medical [2,3] and systems improvement literature, as summed up in the adage "we improve what we measure"[4,5].

Several areas of measurement and improvement are well researched, particularly in areas of care for chronic conditions [6,7], implementation of information technology in clinical care settings [8,9], and the impact of formal learning collaboratives [10-12]. There is further evidence that financial incentives, real and potential, affect the provision of care,[13-16] as may unique interactions provided at the small-team or microsystem level [17-19]. Quality improvement activities and the incorporation of systems (e.g., information systems, care management, work flow redesign) in large group practices (>20 physicians) are also well studied, [20-22] and highlight the resources and approaches needed to overcome barriers to quality improvement, including a lack of staff, resources, and time [20-22].

Despite these advances, however, the practical application of measurement and innovation to enhance quality in practice requires substantial motivation and resources, and may be difficult to implement and maintain, particularly in small office settings. Increased burden, lack of infrastructure, and unsophisticated or lack of health information technology have all been reported as significant challenges to performance measurement in small offices [23,36]. Moreover, physicians in small office settings may possess a limited, and highly variable, understanding of quality improvement [24].

Compared to large group practices, small practices may also face unique challenges due to limited resources, particularly to limited staff. For example, medical record reviews may impose substantially higher burdens on smaller practices, simply because there are fewer employees to do the work [23]. Further, though claims data may not impose an additional burden on physicians themselves, the data may be subject to greater errors since it may be difficult to attribute patient care to a smaller practice due to care delivered externally. In some cases, health plans give physicians opportunities to correct these errors, but in many small practices, a lack of adequate staff to perform the required activities hinders the QI activity.

Some research suggests a significant positive correlation between practice size and the use of systems positively impacting quality of care [20]. Presumably, then, small practices would yield the lowest rate of adoption of systems related to QI [20]. Because approximately 75% of primary care is provided in practices consisting of 10 or fewer physicians, optimizing the adoption of quality improvement and measurement approaches in these practices is critical to initiatives to enhance the quality of care in the U.S.[25]. This increased awareness may help identify policies that facilitate QI in small practices, thereby increasing the likelihood that positive health outcomes will follow.

To this end, this study explores factors that hinder and/or facilitate the adoption of quality improvement in small practices that are actively engaged in measurement or improvement activities. It describes *how and why *small practices are successful in the adoption of QI, and presents a framework from which others may build on.

## Methods

### Design and sample

We undertook a qualitative study, based on semi-structured, open-ended interviews conducted with practices that used performance data to drive quality improvement activities. Qualitative methods are well suited to explore substantive areas about which little is known, and often used to obtain details that are difficult to extract or learn about through more conventional research methods [26,27]. Given the paucity of data about performance measurement and QI in small practices, and the anticipation that some factors may be difficult to conceptualize and measure, qualitative, open ended interviews were a suitable choice for this topic [24].

Convenience based purposive sampling was used to obtain a diverse spectrum of specialties, regions, group sizes, and improvement domains (e.g., disease management, open access scheduling, electronic medical records). Participants were recruited through a variety of methods: First, an advisory panel assisted the project team by supplying references based on professional contacts and membership databases. Subsequently, physicians selected for inclusion were queried for referrals to colleagues who fit the study's criteria. Finally, the advisory panel advertised the study through e-mail, internet, and print communications.

### Data collection

**Figure 1:**
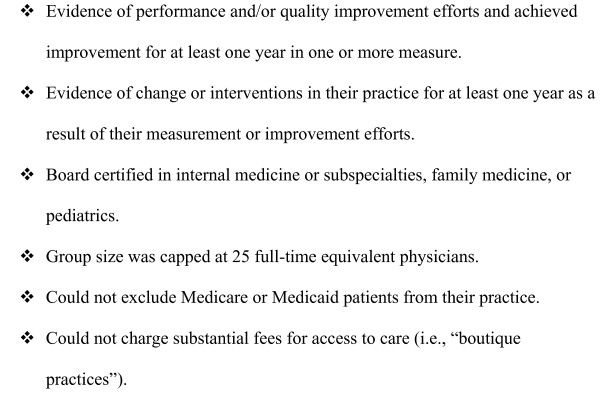
**Physician "adopter" inclusion criteria**.

All eligible candidates were pre-screened by telephone. An interviewer used a detailed guide to determine each physician's eligibility. The intent was not to infer anything about the overall quality of the practice, but rather to determine if clinical performance measurement was routinely used to improve performance in the practice. The full criteria for designation as an "Adopter" can be found in Figure 1.

**Figure 2:**
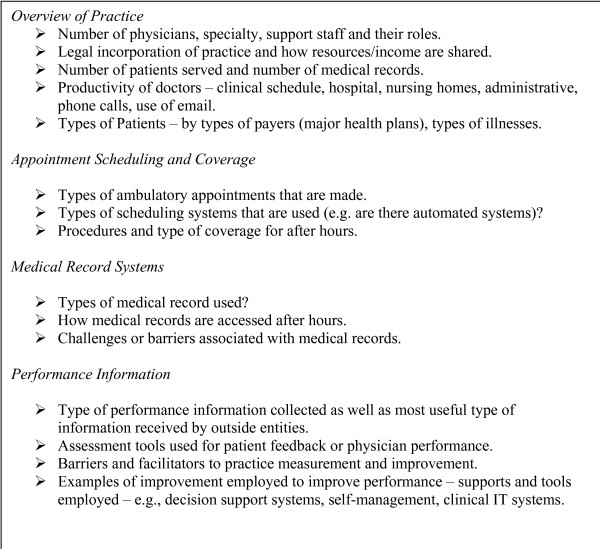
**Outline for interviews**. Additional prompts and probes utilized as necessary to gather detailed information about performance improvement and measurement.

A total of 102 physicians were pre-screened and verbally consented, recruited from the methods above. Pre-screening terminated when data became repetitive. Of these, 39 were chosen for inclusion. The other 63 were not asked to participate due to insufficient evidence of direct involvement in performance measurement and improvement. Where multiple physicians within a group were involved in the practice's improvement activities, we interviewed the physician who professed to have the greatest personal involvement and working knowledge of the group's efforts. Shortly after they were selected for inclusion, physicians were interviewed in person using open – ended questions to allow respondents to answer in their own frames of reference, with minimal influence from the interviewer. The protocol for the interviews can be found in Figure 2.

Each interview was videotaped and lasted about an hour. During each interview, an interviewer facilitated the discussion while a secondary interviewer completed a coding sheet. Each coding sheet indicated the presence or absence of various topics and conditions pertaining to quality improvement. Preliminary codes were derived from the experience and knowledge of experts, as well as existing theory [28,29] and previous research on quality improvement in small office settings. (e.g.[20,21,23]) As data collection progressed, additional codes were identified and added to the coding sheet based on repeated readings of previous interviews. Care was taken in the creation of the coding guide to ensure that it was an iterative and flexible process, and to include categories that represented all relevant aspects of the construct, were mutually exclusive, and had clear definitions, easy to follow instructions, and unambiguous examples. These features enhanced coding reliability, both across different coders and over time [27].

Shortly after each interview, the interviewers watched the videotape to verify the data collected, and a written report was created. The report and the coding sheet were later shared with the participating physician, and feedback was incorporated into the report to ensure the interviewer interpreted the encounter as accurately as possible.

### Data analysis

Content analysis was used to analyze the data [28]. Since codes were established a priori, content analysis was a fitting choice as it is deductive in its approach, and has as its objective to test hypotheses, not develop them [30,31].

After coding, several steps were undertaken. First, we produced summaries of the frequencies of the codes. Second, we examined the patterns and relationships among our findings, and finally, we attempted to relate these higher level themes to those in other situations or in previously reported studies. Our analytic goal was to depict the "big picture" of quality improvement in small practices, though we also display conceptual depth by providing quotes in support of key themes.

## Results and discussion

### Sample characteristics

Forty-two percent (N = 18) of participants were general internists; the rest specialized in family medicine (N = 12) and pediatrics (N = 7). A small percentage (N = 2; 5%) reported other medical subspecialties. The median physician practice size was 6.0, and six were solo practitioners. The majority (almost one-third) worked in physician offices, 10% worked in hospital outpatient clinics, and almost 8% worked in designated public health centers. Twenty-one percent of the physicians were women (see Table 1 for full list of demographics).

**Table 1:** Physician demographics (N = 39)

	Percentage		Percentage
**Physician Specialty**		**Practice Location**	

Internal Medicine	N = 18; 46%	Freestanding	N = 23; 59%

Family Practice	N = 12; 31%	Office Building	N = 9; 23%

Pediatrics	N = 7; 18%	Hospital Outpatient Clinic	N = 4; 9%

Med. Subspecialty	N = 2; 5%	Health Center	N = 4; 9%

**Practice Size (FTE)**		**Year Graduated Medical School**	

Mean	7.2	1961–1970	0

Median	6.0	1971–1980	N = 16; 42%

Solo	N = 6; 15%	1981–1990	N = 17; 47%

2–5	N = 12; 15%	1991–2000	N = 6; 11%

6–10	N = 12; 31%		

11–25	N = 9; 23%		

**Region**		**Information Support**	

New England (MA, ME, NH, VT)	N = 10; 25%	Paper medical records	N = 23; 61%

Southeast (GA, FL, NC, SC)	N = 8; 21%	Electronic medical records	N = 15; 39%

Great Lakes (IL, MI, MN)	N = 6; 15%	Paper registries	N = 4; 10%

Mid-Atlantic (DE, MD, NJ, PA)	N = 5; 13%	Electronic registries	N = 13; 34%

Southwest (CO, TX)	N = 5; 13%		

Northwest (OR, WA)	N = 5; 13%		

### Findings

After initial review of the data, codes were organized into three broad themes for classifying physicians' experiences with quality improvement: perceived benefits of measurement and practice improvement, facilitators, and barriers to quality improvement. Using this framework to organize the data guided analysis *within *each practice as well as *between *practices. Importantly, findings illustrated that small practices have the ability to succeed at quality measurement and improvement, but that there are varying degrees of success given available system and practice resources.

#### Perceived benefits of practice improvement

##### Physicians reported greater efficiency, clinical staff and patient satisfaction/retention, and improved practice reputation as a result of QI activities

Seventy-four percent of physicians noted unanticipated improvements in efficiency and standardization, and 71% noted higher levels of satisfaction in both patient satisfaction and retention. Many (over a third) described improved physician-patient communication channels, patient education efforts, and coordination of care (Table 2). As one physician reported,

**Table 2:** Perceived benefits of quality measurement and improvement on practice and patients

**Benefits for Practice**		**Benefits for Patients**	
Efficiency, standardization	N = 29; 74%	More appropriate, effective care	N = 33; 84%

Patient retention/satisfaction	N = 28; 71%	Improved timeliness of care	N = 26; 66%

Improved reputation	N = 26; 66%	Patient safety	N = 18; 45%

Clinical staff retention/satisfaction	N = 23; 58%	Access to information	N = 15; 39%

Improved patient outcomes	N = 20; 50%	Communication with physicians	N = 14; 37%

Improved revenues	N = 15; 39%	Care coordination	N = 13; 34%

Support staff retention/satisfaction	N = 11; 29%	Access to care	N = 11; 29%

Ability to satisfy external requirements	N = 7; 18%	Interaction with other staff	N = 11; 29%

Factors cited as benefits of quality measurement and improvement, reported in percentages of physicians who reported them.

"Since we implemented our quality indicators and our quality mechanisms, everything has changed: our attitude, our communication internally, because we are always looking for new ways of doing things. People come to us all the time and say "why don't we measure this or that? ...it's always renewing.(Oncologist)"

Approximately 40% of the practices reported increased revenues as a result of their internal QI initiatives, and no physicians reported decreased revenues. Finally, 66% reported feeling that their reputation in their community had improved, and over half reported enhanced patient outcomes. This link was articulated nicely by one physician,

"We took our cycle time from an average of 80 minutes... (that's not something that you would be very proud to put out there)... to just over 30 minutes. It also dramatically improved our bottom line. If you see more patients and you do it more efficiently the patients are more satisfied, and that's one of the things that makes these initiatives important to us, because we see that we can make significant change (Pediatrician)"

##### Physicians do not rely on financial incentives to motivate them

Financial incentives were not a major motivating factor, as only 13% reported using them for intrinsic motivation to QI. More frequently, a physician leader or a team of leaders reacted to evidence of sub-optimal performance as indicated by data they had collected themselves or received from a trusted external source (Table 3). These leaders expressed a desire to understand the data and were then driven primarily by intrinsic professional motivations to seek strategies to raise performance levels. Whereas many physicians expressed concern about external data sources, they responded by checking the data themselves or supplementing the information with their own studies. Though less frequently expressed, additional motivators included the notion of benchmarks and public reporting,

**Table 3:** Motivators to measure and improve

**Motivators to Measure**		**Motivators to Improve**	
Identification of problem by practice leader	N = 23; 58%	Identification of problem by practice leader	N = 28; 71%

Training in quality measurement or improvement	N = 16; 42%	Available solution that appeared feasible	N = 21; 55%

Available product or tool	N = 12; 32%	Trends evident in data	N = 20; 53%

Encouragement from colleagues, societies	N = 8; 21%	Comparing performance to benchmarks	N = 15; 39%

Dissatisfaction/loss of clinicians	N = 7; 18%	Examination of trends by colleagues	N = 12; 32%

Identification of problem by consultant	N = 6; 16%	Identification of problem by consultant	N = 9; 24%

Financial incentives	N = 5; 13%	Financial incentives	N = 7; 18%

Pressures from plans, purchasers, etc.	N = 5; 13%	Dissatisfaction/loss of clinicians	N = 6; 16%

Dissatisfaction/loss of patients	N = 2; 5%	Pressures from plans, purchasers, etc.	N = 3; 8%

Publication of report on safety, errors	N = 2; 5%	Publication of report on safety, errors	N = 3; 8%

Dissatisfaction/loss of support staff	N = 1; 3%	Dissatisfaction/loss of patients	N = 3; 8%

Malpractice losses/increased premiums	N = 0	Dissatisfaction/loss of support staff	N = 1; 3%

		Initiation of public reporting	N = 1; 3%

Factors cited as motivators to quality measurement and improvement, reported in percentages of physicians who reported them.

"We set ourselves up as a small group in primary care practice and knowing that we will be reported in a public way is a real powerful motivator (Family Practitioner)"

as well as the provision of data showing gaps between ideal and actual performance:

"The first query we did we found that we treated women with lung cancer less aggressively than we did men. So we came back and discussed that and changed our behavior all together. It was a real eye-opening experience that has created actionable knowledge. It wasn't something that we wanted to see but it was something that was very important for our patients (Oncologist)."

#### Facilitators to quality improvement

##### Leadership and teamwork are critical to performance improvement

Leadership emerges repeatedly as a necessary prerequisite to change (Table 4). Consistent with previous literature, more than half the physicians cited practice leadership as crucial to the initial measurement activity, while almost three-quarters point to the role of leadership and cooperation in implementing a QI strategy [32-35]. As reported by many physicians, leaders are "instrumental in deciding to pursue QI", in that they initiate performance assessment, commit resources to new strategies, achieve buy-in from colleagues and staff, build a culture supportive of QI, participate in learning collaboratives, and engineer office practices to sustain improvements. However, leaders do not work in isolation – almost half (45%) of physicians reported the 'teamness' of the practice as an internal facilitator to QI. One physician noted that his staff "*feels like they can point at our systems and recognize where they made a contribution", and by "distributing that and by capitalizing on all the expertise that builds up in our group, no one individual is overburdened by it*".

**Table 4:** Facilitators to quality measurement and improvement

**Internal Facilitators**		**External Facilitators**	
Idea champion	N = 28; 71%	Learning collaborative	N = 14; 37%

Cooperation of physicians	N = 28; 71%	External funding or assistance	N = 12; 32%

Commitment of physician/team	N = 26; 66%	External vendor or consultant	N = 9; 24%

Cooperation of other clinical staff	N = 26; 66%	Lack of external pressure	N = 8; 21%

Cooperation of support staff	N = 23; 58%	Support from plans, purchasers	N = 7; 18%

Investment of time, energy by leadership	N = 21; 55%	External pressures	N = 7; 18%

"Teamness" of the practice	N = 18; 45%	Financial incentives	N = 6; 16%

Available resources	N = 18; 45%		

Systems to track progress	N = 12; 32%		

Cooperation of patients	N = 5; 13%		

Factors cited as facilitators to quality measurement and improvement, reported in percentages of physicians who reported them.

This sense of empowerment is further noted in one physicians' account of how his staff contributed:

"We post unblinded measurement, both comparative with other clinics and individual provider measurements on different things in our break room. It motivates a medical assistant who worked with a physician provider or nurse practitioner provider to think, how can I get my provider's numbers better? What am I doing that might help? Here, you bring in not just the professional energies and competition of the providers, but you really distribute the professional satisfaction around to everybody else in the group. And once everybody is on board the professional energy that results make it a lot easier than you expect it to be when you start out (Family Practitioner)"

Further, practices that succeeded in QI have office cultures that value teamwork and shared responsibility, and feature routine, matrixed interaction among physicians and between doctors and support staff, "*instead of the physician being at the center of everything and everything moving around the staff"*. New tasks related to changes are often delegated to staff that previously were not involved in more direct clinical aspects of care, with positive results. One physician describes this process:

"The staff became part of the implementation – what kinds medicines would be used, what kind of work flows we would have. So each one of them had a little something that they were promised they would be getting out of this project. We found that after a week the patient demand was already so great that we were seeing patients just like we had before and we were electronic (Internist)"

In this context, physicians spoke of their practices' internal agreement on "collective values" and clear delineation of responsibilities within their teams. Though teamwork was essential, implementation was usually led by an improvement "champion," who was typically not the practice owner or manager, and who involved a mix of physicians and support staff in designing and effecting change.

##### External Facilitators include learning collaboratives and external assistance

Physicians worked at developing competencies in systems thinking, quality improvement techniques, team building, and data analysis. In more than a third of these practices (37%), these skills were developed with the assistance of medical societies or learning collaboratives, such as the Institute for Healthcare Improvement (IHI) in Boston, and the Institute for Clinical Systems Integration (ICSI), which provided education on quality improvement techniques and guidance in developing and implementing practice changes. The internet also served as a valuable resource for information about quality of care, and some physicians joined listservs or other online discussion groups devoted to sharing information about QI. Even with external assistance, however, clear and attainable goals were important:

"If you don't have clear aim, if you can't state in a sentence or two what it is that you want to do, you're never going to be able to bring somebody along with you...we tried to figure out what the messages were that would bring other people on board which would then help us with our overall project (Internist)."

##### Decision support is a fundamental building block of better clinical care, yet successful QI initiatives do not require expansive technologic interventions

While 17 physicians had disease registries, low-cost and low-tech solutions were more prevalent than expensive technologic interventions [36,37]. Approximately half used electronic or paper registries rather than full electronic medical records to collect information (usually from flowsheets) on individual patients and provide real-time feedback on patients' clinical status and tests performed. Other low-cost changes included issuing standing orders, reorganizing staff responsibilities, and enhancing patient education efforts.

##### Success breeds success

Improvement often starts with a single effort to improve one aspect of care, but demonstrable success initially provides encouragement for additional activity and helps secure the support of colleagues who may have been initially resistant.

One physician reported that,

"By having everyone thinking quality improvement, clinical improvement – just using your focus and your energy to improve quality, by creating that kind of culture it makes it a lot easier to get the right thing done (Multi-specialist)"

A number of physicians reported that QI gains momentum with each new effort, and processes are modified in ways that facilitate additional changes. Creating a culture amenable to QI is essential, and involves changing attitudes about QI as well as available resources. In sum, integral to improvement in these practices was not the technological changes, but rather, cultural and leadership changes that were dependent on efficient leadership, cohesiveness of the group, and a strong sense of teamwork among the staff.

#### Barriers to quality improvement

Contrary to prior research, small practice size was not frequently cited by physicians as a barrier in implementing changes. In fact, several physicians noted that small practice size was an advantage in that it mitigates the need to gain buy-in from many different participants, permits greater flexibility, and facilitates the formation of a cohesive microsystem.

However, physicians did encounter barriers during their attempts to improve (Table 5). Sixty-six percent cited time as a major challenge to implementation, and many complained that it sometimes took 2 to 3 years before they saw evidence of improved outcomes. Here, effective leadership and cultural buy-in are critical to success. Improvement may not necessarily require a large investment of time at any given point, but rather a willingness and ability to persevere over a long period of time.

**Table 5:** Barriers

**Barriers**		**Impact of Barriers**	
Time constraints	N = 26; 66%	Changed timeline	N = 12; 31%

Costs	N = 18; 47%	Provided training	N = 8; 21%

Equipment problems	N = 11; 29%	Changed staffing	N = 5; 13%

Lack of properly trained/motivated staff	N = 10; 26%	Brought in external consultants	N = 2; 5%

Took more time than expected	N = 9; 24%	Changed systems/processes	N = 2; 5%

Lack of financial incentives	N = 8; 21%	Increased budget	N = 1; 3%

Resistance of clinical staff	N = 7; 18%	Changed leadership	N = 0

Staff turnover	N = 5; 13%		

Process more difficult than expected	N = 4; 10%		

Conflicting pressures from others	N = 4; 10%		

Used more resources than expected	N = 3; 8%		

Incompatible systems/processes	N = 3; 8%		

Evaluation of results	N = 2; 5%		

Tracking implementation	N = 2; 5%		

Lacked information on process	N = 1; 3%		

Lack of nonfinancial rewards	N = 1; 3%		

Factors cited as barriers to performance measurement and quality improvement, reported in percentages of physicians who reported them.

One critical finding is that the smallest practices (10 or fewer physicians) rarely focused on more than two clinical areas. Even those with access to a wide array of measures through electronic medical records were severely hampered by time and resource constraints when they tried to improve in more than one or two domains. In other words, while these small practices were "successful" at quality improvement, the process took substantial time, and there appeared to be limits on what small practices can accomplish at any given time.

Almost half of the physicians reported cost as a major barrier to implementation (47%), which is consistent with prior research that found that small practices cite cost as the main reason for not adopting electronic health records [38]. Some physicians acknowledged that over time, costs decreased as other benefits emerged:

"I think a lot of doctors see the cost as something that comes out of their pocket rather than costs that are amortized over a period of time; and also, costs that help to make the office more efficient so that you practice better, and your costs go down, and then the numbers of patients that you can see and do a good job with are better" (Internist)

Additional barriers cited included equipment problems (29%), lack of properly trained or inexperienced staff (26%), and other problems with staff (i.e. turnover, resistance; 13%). Interestingly, only a small number (8%) reported incompatibility with their systems or processes, while only 3% noted a lack of information of the QI process.

## Conclusion

This study examined the perceived benefits of utilizing QI methodologies, as well as what factors inhibited or facilitated practice improvements. Interestingly, small practice size was not frequently cited as a barrier to quality improvement. Often practices were motivated by a professional drive to close a gap in performance (optimal care versus actual care delivered) discovered once a performance assessment was conducted and data was available for review. Many of these physicians felt that external resources were integral to their learning about practice systems and leading change within their practices.

The findings revealed some unique patterns, particularly regarding the relative unimportance of financial incentives (and extrinsic motivators) on the one hand, and commitment to improve quality (and intrinsic motivators) on the other. Physicians did not identify financial incentives among the five most important motivating factors in the adoption of performance improvement efforts, however, cost was the third most frequently barrier cited. Further, findings highlighted the smallest practices can only focus on one, or perhaps two, QI initiatives at any one time. Future research should investigate the effect of implementing one or two QI initiatives in these practices: is there a positive, or negative, spillover effect? If the system change is broad in scope, then conceivably other aspects of the practice may improve. However, system changes in one area may also negatively affect other areas of the practice.

The study suggests the interaction of factors; for example, exposure to QI collaboratives, provision of data, and the emergence of a commitment to QI in the practice are important to successful QI efforts. Physicians were not deterred by the barriers they faced, even when they found the initial data to be imperfect or a substantial performance gap. Some of the physicians' motivations appeared to stem from a strong professional commitment to performance improvement [39,40]. Finally, findings suggest professional societies and training institutions can be effective in inculcating the core values of professionalism and systems-based thinking with existing physicians, students and residency level trainees by emphasize that continuously working on practice improvement is a key element of professionalism.

One of the most compelling findings is the seemingly high level of satisfaction with which physicians approach their work. Many studies have shown a correlation between physician dissatisfaction and a loss of autonomy, increasing responsibility, changing reimbursement arrangements, and heightened expectations of patients, payers, insurers, and regulators,[41-44] so it is worth noting the invigoration these physicians describe. Whether this trait of optimism was preexisting, or acquired as a result of their involvement in quality measurement and improvement or some other opportunity, is a critical factor to explore in more definitive studies. The results emulate the blueprint described by Mechanic [45] for infusing physicians with renewed energy and fulfillment by focusing on management of information, treatment of chronic conditions, and redesigning of office work patterns.

In summary, creating and sustaining a broad based quality improvement effort in small practices looms as one of both the primary challenges and the primary opportunities in achieving the aims of the Institute of Medicine. Based on our findings, one could argue that a robust national effort to improve quality will require attention to both intrinsic (professionalism) and extrinsic (incentives) approaches. The balance of intrinsic and extrinsic rewards needs to be more fully understood. Insurers and government agencies evaluating the potential impact of financial rewards should consider the lessons of these small groups as well as the resources and competencies they require.

The role of support within the profession, such as embracing a professionalism ethic that espouses quality improvement as a central tenet, and the role of learning collaboratives also appear critical to small practices through their provision of expertise, resources, measurement tools and most importantly, motivation of individual physicians. National organizations may have a vital role to play in the extension of quality improvement activities, and additional research is warranted on the precise aspects of support that proves most useful for physicians.

There were several limitations to this study. First and foremost, we acknowledge a larger conundrum in quality improvement more generally; that is, that measurement alone does not necessarily translate into meaningful differences in patient's lives [46]. In other words, 'success' in quality improvement in small practices may not translate to better patient outcomes in all instances, and more work is needed to tailor clinical care and measurement to individual patients rather than using a mechanistic approach [46].

Methodologically, our qualitative sample was purposive and aimed not at being representative. The results should be interpreted as suggestive rather than conclusive. We acknowledge that the large number of practices excluded from the study may have systematically differed from those chosen, and our sample may therefore reflect only physicians and practices amenable to QI. As a result, our findings may only be applicable to practices whose cultures and physicians are fond of measuring QI. We agree that more nuanced qualitative research exploring practices that were less successful in building QI activities, and why they were unsuccessful, is needed. Lastly, our conceptualization of small practices may not be particularly relevant in Europe where a practice of more than 5–7 physicians would be considered quite large, and as such, our results should be interpreted as generalizable to U.S. small practices only.

Further, because many of our codes were generated a priori, we acknowledge the potential for preconceived notions about the data. This is a frequently reported dilemma in research: many argue that theories inform research in multiple ways, even if used un-self-consciously [47]. In this study, using codes may have biased the way we observed the data through our own perspectives, shaping our observations of physician behavior and potentially omitting novel information raised in the interviews. However, we sought to minimize this bias by making the coding an iterative process, adding new codes when introduced by the data, as well as by using two interviewers.

Despite these limitations, the present study raises several key areas for future research on QI in small practices to explore, and provides a framework on which others may build. An important challenge for dissemination will be to explore how these data can be more readily and practically transported into the clinical setting, given the practical and financial barriers.

## Competing interests

This research was funded by the Commonwealth Fund, New York, NY and the ABIM Foundation, Philadelphia, PA. Funders provided assistance in design of the study and review of the manuscript.

## Authors' contributions

DW helped conceptualize and fund the design, conducted the interviews, and provided valuable insight into the drafting of the manuscript. BL conducted the interviews and participated in data analysis. SS aided in the conceptualization of the design and analysis, and facilitated the drafting of the manuscript. GP participated in the design of the study and drafted the manuscript. DP participated in study design and coordination and helped to draft the manuscript. EB participated in the data analysis, and compiled authors' drafts, and is responsible for the final draft of the manuscript. All authors read and approved the final manuscript.

## Pre-publication history

The pre-publication history for this paper can be accessed here:


